# Editorial: Decoding and bridging the tripartite components of One Health: collaborative strategies for global well-being

**DOI:** 10.3389/fpubh.2026.1815200

**Published:** 2026-03-25

**Authors:** Christos Stefanis, Christina Tsigalou, Elisavet Stavropoulou

**Affiliations:** Laboratory of Hygiene and Environmental Protection, Department of Medicine, Democritus University of Thrace, Alexandroupolis, Greece

**Keywords:** AMR, civil protection, climate change, digital health, diseases, education, environmental health, One Health

## Introduction

The One Health approach is increasingly recognized as essential for understanding contemporary global health threats. Given the close interconnections among humans, animals, and ecosystems, this perspective facilitates the effective addressing of major challenges such as the climate crisis, emerging infectious diseases, social instability, and environmental degradation.

This Research Topic presents studies that demonstrate the breadth and interdisciplinary nature of the One Health approach. Topics include waste management, environmental sustainability, pandemic preparedness, and mental health. Collectively, these studies illuminate the interconnections that influence both human populations and ecosystems and propose strategies to strengthen health systems.

## Environmental challenges and antimicrobial resistance

Inadequate waste management results in environmental degradation and poses significant public health risks. One study in this Research Topic addresses this concern. Petropoulou emphasizes that improper management of solid and liquid waste adversely affects public health across all communities, not solely the most vulnerable. These impacts arise from the proliferation of pathogens and the increasing prevalence of antimicrobial resistance.

Sams-Dodd and Sams-Dodd argue that antimicrobial resistance is not solely a consequence of climate change. It also disrupts the balance of microbial ecosystems and further exacerbates climate change.

## Sustainability and social wellbeing

Interdisciplinary collaboration within the One Health framework extends beyond immediate public health threats. It encompasses sustainable food systems and circular economy principles. These approaches integrate human, animal, and ecosystem health from a complementary perspective.

Katsafadou et al. integrate these dimensions using the olive tree as a focal point. Their study connects cultivation, processing, health benefits, agricultural practices, and by-product management. This approach demonstrates that One Health extends beyond risk mitigation, promoting sustainability and supporting resilient social systems centered on both human and environmental wellbeing.

In addition to interactions with plant ecosystems, contact with animals may also benefit human health. Tang et al. demonstrate that regular interaction with companion animals positively influences wellbeing indicators, including sleep quality among older adults. Thus, human wellbeing can arise from interactions with plants, animals, and the environment, reflecting a core principle of the One Health approach.

## Governance and crisis preparedness

The vulnerability of specific population groups, particularly children, is emphasized in the article by Masetti et al.. The study investigates how climate change, antimicrobial resistance, and emerging infections disproportionately impact children, a vulnerability linked to physiological immaturity and critical developmental stages. In low- and middle-income countries, safeguarding children's health necessitates multidimensional strategies within the One Health framework.

Preparedness for future respiratory pandemics is closely tied to equity in global research capacity. Hossain and Kim contend that lung organoids serve as essential human-relevant models, facilitating rapid pathogen study and therapeutic evaluation. The authors also underscore disparities in access to these technologies. Enhancing research equity and building capacity before crises is crucial, while fostering trust remains vital for collective resilience and an effective response.

Martinez-Hollingworth et al. examine the human dimension of environmental crises, demonstrating that frontline workers serve as both care providers and individuals directly impacted by disasters. This dual role highlights the necessity for policies that support mental health and professional resilience, as well as the importance of collective wellbeing during complex emergencies.

Two additional studies emphasize the significance of institutional structures and governance networks (Saidouni et al.; Togami et al.). In contexts characterized by social and political instability, intersectoral collaboration enhances preparedness. Cooperation among public health, veterinary, and environmental sectors fosters stability and progress. Trust and transparency between institutions and state actors are critical, underpinning effective preparedness and response, particularly during pandemics.

In conclusion, the studies included in this Research Topic illustrate that One Health serves as both a holistic framework and a strategic roadmap. It integrates actions that address complex, interacting determinants of health, aiming to strengthen resilience, build trust, enhance governance, and safeguard the health of future generations.

